# Hyaluronic Acid‐Based 3D Bioprinted Hydrogel Structure for Directed Axonal Guidance and Modeling Innervation In Vitro

**DOI:** 10.1002/adhm.202402504

**Published:** 2024-11-06

**Authors:** Laura Honkamäki, Oskari Kulta, Paula Puistola, Karoliina Hopia, Promise Emeh, Lotta Isosaari, Anni Mörö, Susanna Narkilahti

**Affiliations:** ^1^ Neuro Group Faculty of Medicine and Health Technology Tampere University Tampere 33520 Finland; ^2^ Eye Regeneration Group Faculty of Medicine and Health Technology Tampere University Tampere 33520 Finland

**Keywords:** axon orientation, human pluripotent stem cell‐derived neurons, hyaluronic acid, multi‐material 3D bioprinting

## Abstract

Neurons form predefined connections and innervate target tissues through elongating axons, which are crucial for the development, maturation, and function of these tissues. However, innervation is often overlooked in tissue engineering (TE) applications. Here, multimaterial 3D bioprinting is used to develop a novel 3D axonal guidance structure in vitro. The approach uses the stiffness difference of acellular hyaluronic acid‐based bioink printed as two alternating, parallel‐aligned filaments. The structure has soft passages incorporated with guidance cues for axonal elongation while the stiff bioink acts as a structural support and contact guidance. The mechanical properties and viscosity differences of the bioinks are confirmed. Additionally, human pluripotent stem cell (hPSC) ‐derived neurons form a 3D neuronal network in the softer bioink supplemented with guidance cues whereas the stiffer restricts the network formation. Successful 3D multimaterial bioprinting of the axonal structure enables complete innervation by peripheral neurons via soft passages within 14 days of culture. This model provides a novel, stable, and long‐term platform for studies of 3D innervation and axonal dynamics in health and disease.

## Graphical abstract

Multi‐material 3D bioprinting of two hyaluronic acid‐based bioinks with different stiffnesses is used for 3D axonal guidance structure in vitro. The used bioinks are mechanically characterized and printed as alternating, aligned filaments. A successful, complete innervation with human neurons is achievable within the structure.

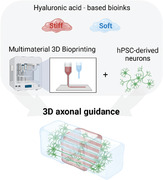

## 1 Introduction

The human nervous system, which consists of the central nervous system (CNS) and peripheral nervous system (PNS), possesses extremely complex physiology. Together, the CNS and PNS are responsible for the coordination of all voluntary and involuntary activities in humans.^[^ ^1^, ^2^ ^]^ For example, PNS neurons elongate long axons to innervate all the tissues in the body.^[^ ^3^ ^]^ Axonal damage due to disease or trauma causing disruption of neuronal communication leads to the loss or impairment of crucial functions. The CNS has a low intrinsic regenerative capacity after injury,^[^ ^4^ ^]^ and while PNS has better regeneration potential, spontaneous recovery is limited to only small distances.^[^ ^5^ ^]^ Animal models are still widely used in neural tissue engineering (TE); however, they often fail to recapitulate the physiology and pathophysiology of, e.g., the complex human nervous system leading to failures in clinical trials.^[^ ^6^, ^7^ ^]^ Moreover, innervation is often overlooked in TE applications despite its pivotal role in the development, maturation, regulation, and regeneration of tissues and organs.^[^ ^3^ ^]^ Thus, increasing effort has been put into developing human cell‐based artificial 3D TE constructs to understand the functions of the human nervous system in health, disease, and injury.

Neuronal tissue has an organized, distinct architecture composed of neurons, supporting neuroglial cells, and extracellular matrix (ECM). The specific 3D organization of the cells and the ECM structure of the neuronal tissue varies depending on the location in the body.^[^ ^8^ ^]^ However, the axonal pathways in both the CNS and the PNS share an aligned and unidirectional architecture of axon fascicles.^[^ ^2^, ^9^ ^]^ Axonal organization during development and after injury relies on the multifarious responses of axons to extracellular biophysical and biochemical guidance cues.^[^ ^10^, ^11^ ^]^ The biophysical cues of the ECM, including elasticity and structure, as well as surface topography, are known to affect cellular behavior such as controlled neuron migration, nerve regeneration, and axon growth.^[^ ^12^ ^]^ The brain ECM consists of glycosaminoglycans, proteoglycans, glycoproteins, and low amounts of proteins. The main structural component is hyaluronic acid (HA), and proteins such as collagens and laminins are also components of the ECM in both the CNS and the PNS.^[^ ^13^ ^]^ They have all been utilized in neuronal TE applications in vitro.^[^ ^14^ ^]^ Biochemical cues have either attractive or repellent effects on axonal growth. The guidance molecules in both the CNS and PNS include netrins, slits, ephrins, semaphorins, a family of neurotrophins, and various growth factors, such as nerve growth factor (NGF).^[^ ^15^ ^]^ Moreover, axons use contact guidance cues offered by other cells^[^ ^13^, ^16^ ^]^ and tissue structures, e.g., the vasculature,^[^ ^17^ ^]^ for aligned axon growth. Thus, to achieve directed axonal growth in 3D, natural‐like guidance cues need to be included in the engineered structure, which is challenging with conventional fabrication techniques and homogeneous counterparts.^[^ ^18^, ^19^ ^]^ Approaches such as electrospun fibrous scaffolds,^[^ ^20^, ^21^ ^]^ aligning self‐assembled ECM proteins with microfluidic flow,^[^ ^22^ ^]^ composite structures of hydrogels and electrospun nano/microfibers^[^ ^23^, ^24^, ^25^ ^]^ and orienting short fibers in hydrogels^[^ ^26^ ^]^ have been utilized for aligned topographies within 3D matrices for neuronal guidance. They, however, possess some challenges, such as a complex scaffold manufacturing process, nontransparent scaffolds, and rigidity that hinders neuron growth and induction of damage.^[^ ^24^ ^]^

3D bioprinting enables automated, repeatable fabrication of complex tissue structures according to predesigned 3D models with high precision spatial positioning of cells, biomaterials, and bioactive factors that mimic the dynamic properties of human tissues and thus has become a vital technique for TE applications.^[^ ^27^ ^]^ Bioink creates a supportive microenvironment for cellular growth and function and needs to meet the demands of bioprinting technology and provide structural integrity to the designed scaffold.^[^ ^28^, ^29^ ^]^ Polymeric hydrogels are often used for neuronal tissue applications because of their tunable mechanical properties and ability to mimic the natural, soft neuronal ECM composition.^[^ ^29^ ^]^ Natural, soft polymers (HA, gelatin, collagen, alginate, chitosan, fibrin, laminin, and decellularized ECM)^[^ ^27^, ^30^ ^]^ have been utilized as 3D scaffolds for neurons, whereas synthetic, stiffer polymers (PLGA, PLA, and PCL)^[^ ^30^, ^31^ ^]^ polymers have been used in supporting structures. HA is one of the most common polymers used in various TE applications as it can be chemically modified and enzymatically cleared in vivo.^[^ ^32^ ^]^ Its initially poor mechanical properties are easily improved with different functional groups, such as polydopamine which is a synthetic polymer derived from dopamine (DA). DA is traditionally considered a neurotransmitter but has recently been shown to enhance scaffold stability and facilitate entrapment on cell‐secreted laminin and growth factors stimulating hydrogel's remodeling properties.^[^ ^33^ ^]^ Moreover, DA enhances cytocompatibility and ECM deposition without inducing cell signaling and neuronal differentiation, making it ideal for more physiologically relevant neuronal in vitro applications.^[^ ^34^ ^]^ The recent trend to develop bioinks mimicking native ECM as closely as possible has been implemented by using ECM components, functionalized synthetic polymers, and multicomponent bioinks for extrusion‐based,^[^ ^29^ ^]^ inkjet,^[^ ^35^ ^]^ microfluidic,^[^ ^36^ ^]^ laser‐based^[^ ^37^ ^]^ and stereolithography^[^ ^38^ ^]^ bioprinting. Extrusion‐based bioprinting has the advantage of being a simple, low‐cost technology with the possibility of using multiple bio‐inks with varying viscosities and cell densities simultaneously^[^ ^39^ ^]^ and has already been used in a few neuronal TE applications in vitro and in vivo.^[^ ^27^ ^]^

Here, we utilized a multimaterial bioprinting approach to produce a 3D axonal guidance structure. Two HA‐based bioinks with different stiffnesses were printed as alternating, parallel filaments. The soft bioink incorporated with the attractants NGF and laminin formed passages for axonal elongation of human pluripotent stem cell (hPSC)‐derived neurons, and the stiff bioink provided structural support and contact guidance. The multi‐material bioprinting process and 3D axonal guidance scaffold preparation were first optimized without cells. The effects of the two crosslinking densities of the HA‐based bioink on neuronal growth and their mechanical properties were subsequently characterized. The effects of NGF, laminin, and cell density on enhancing 3D neuronal network formation were optimized in the soft bioink. Finally, the innervation potential of human cortical and peripheral neurons in the formed 3D axonal guidance structure was demonstrated. This novel, stable, and completely human cell‐based axonal guidance structure offers opportunities for long‐term modeling of 3D innervation and axonal dynamics in vitro in health and disease.

## 2 Results

### 2.1 NGF and Laminin Supplemented Medium Increased Formation of the Neuronal Network in the Bulk HA‐DA Bioink

**Figure 1:**
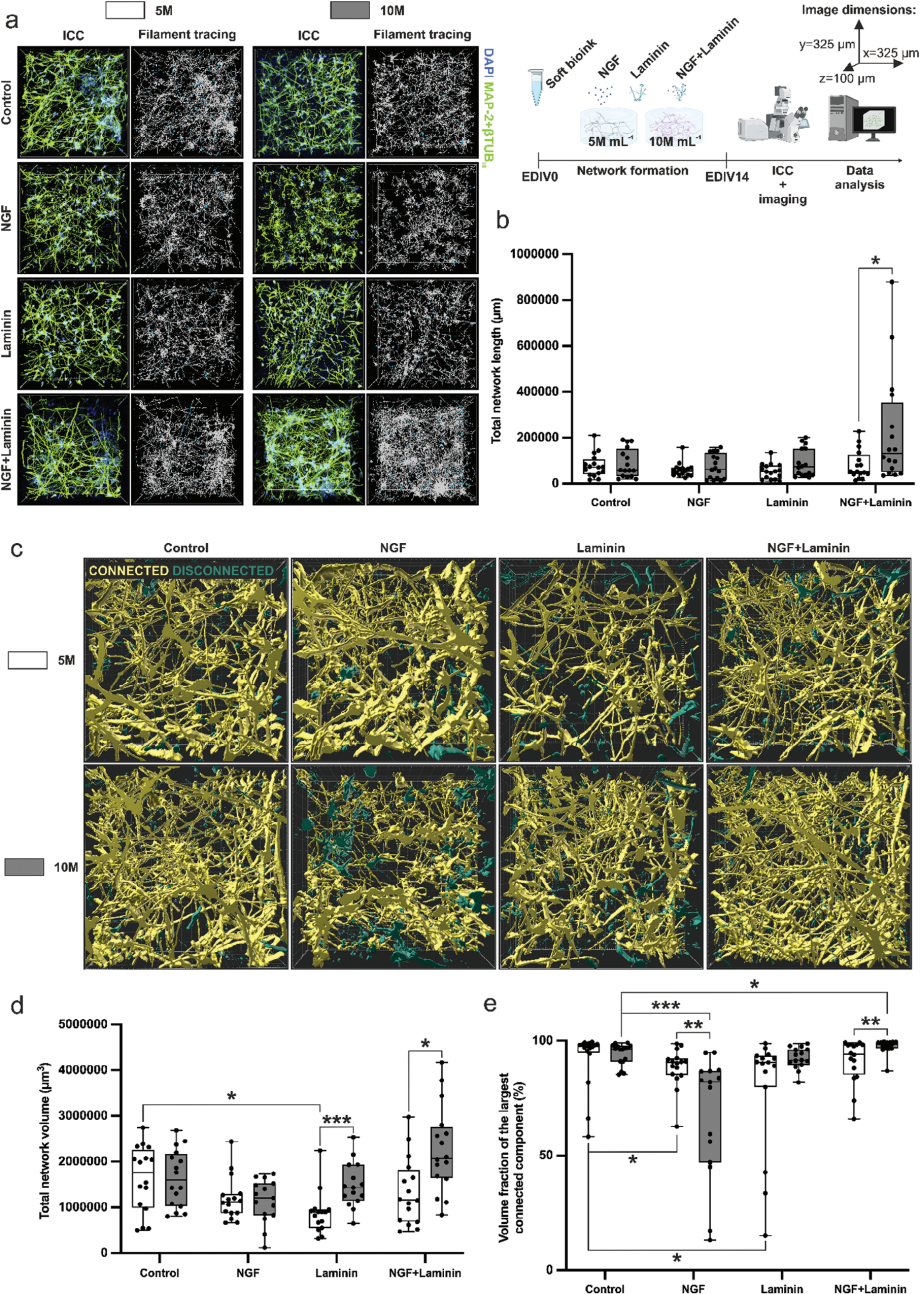
a) 20 × magnification ICC images and filament tracing results for the total network of cortical neurons in 3D bulk bioink cultures at cell concentrations of 5 and 10 m supplemented with NGF, laminin, or NGF+laminin. Neurites are stained with MAP‐2+βtub_III_ (green), while nuclei are stained with DAPI (blue). The timeline and the experimental setup are illustrated in the upper right corner (created with Biorender.com). b) Quantification of the total network length. c) Neuronal network volume and connectivity of the network. Volume reconstructions of neuronal networks with 5 and 10 m cell concentrations for the control groups and cultures supplemented with NGF, laminin, and NGF+laminin. The largest connected component is shown in yellow, and the most disconnected network is shown in green. d) Quantification of the average neuronal network volume and e) quantification of the volume fraction of the largest connected component. The quantifications in b) and d,e) are represented as Tukey box plots with box borders for the 25 and 75 percentiles, whiskers for the maximum and minimum values, and a middle line for the median value. N for each group is 16, resulting in a total of 128 analyzed images from two experiments. Statistical significance is denoted as ^*^ *p* < 0.05, ^**^ *p* < 0.01, ^***^ *p* < 0.001. The image size is 325 µm × 325 µm × 100 µm for all the images.

HA‐based bioink was supplemented with NGF, and mouse laminin, and the two combined to enhance neuronal network formation with the selected cell concentrations. NGF (400 ng mL^−1^)^[^ ^40^ ^]^ and elevated concentrations of laminin^[^ ^41^ ^]^ have previously been reported to increase the neurite length in vitro. 3D neuronal networks were characterized with immunocytochemical staining (ICC) after 14 experimental days in vitro (EDIV) with the neuronal markers MAP‐2 and βtub_III_. ICC images were visualized with Imaris, and the neuronal networks were further quantified using Imaris filament tracing and volume reconstruction tools (**Figure** **1**a,c). In all the groups, the neurons expressed long neurites and dense network formation (Figure 1a). A higher cell concentration, 10 × 10^6^ cells mL^−1^ (10 m), resulted in a trend toward longer total network length in all the groups except the control. The difference was significant in the NGF+laminin group, as the total network length at 10 m reached over 100 000 µm. (Figure 1b)

To further evaluate the networks and the effects of NGF and laminin, total network volume was analyzed from neuronal network reconstructions (Figure 1c). NGF and laminin alone did not increase the total network volume (Figure 1d) or network connectivity (Figure 1e). The average volume fraction of the largest connected component was >80% for all the groups except for the NGF 10 m group, for which it was <70%. The highest connectivity, 98%, was found for the NGF+laminin 10 m group. Thus, NGF+laminin (10 m) performed the best in terms of all the parameters, indicating that supplementing the bioink with NGF+laminin with a cell concentration of 10 m could also be used to increase the axonal growth in 3D printed samples.

### 2.2 Effect of Stiffness on the Formation of the Neuronal Network

**Figure 2:**
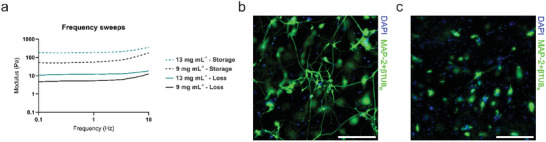
Effect of bioink stiffness on neuronal cell growth. a) Mechanical properties of the soft and stiff bioinks (*n* = 4 for both bioinks). b) Fluorescence images of neuronal cell growth in soft bioink and c) stiff bioink. Scale bars are 200 µm, and the magnification is 20 ×. Neurites are stained with MAP‐2+βtub_III_ (green), while nuclei are stained with DAPI (blue).

Next, the effect of bioink stiffness on neuronal cell growth was studied. First, the mechanical properties of soft and stiff bioinks were evaluated separately via oscillatory rheology. Both the storage modulus (G′) and loss modulus (G″) clearly differed between the soft and stiff bioinks, indicating differences in mechanical strength due to the increased crosslink density in the stiffer bioink (**Figure** **2**a). The soft bioink had a storage modulus of G′ = 71.0 ± 7.3 Pa and a loss modulus of G″ = 6.1 ± 1.1 Pa, and the stiff bioink had a storage modulus G′ = 206.2 ± 19.0 Pa and loss modulus G″ = 12.7 ± 6.9 Pa at 1 Hz. In addition, G′ was greater than G″ at all frequencies; thus, the bioinks behave as viscoelastic solid materials. Neuronal cell growth was affected by the stiffness of the bioink. As shown in Figure 2b, the neurons grew well and extended their processes in the soft bioink, whereas they exhibited a roundish morphology with very few short process extensions in the stiff bioink. Thus, the stiffness difference between the soft and stiff bioink is sufficient for either supporting or preventing neuronal cell growth in the 3D matrix.

### 2.3 Development of an Axon Guidance Structure with Multimaterial Printing Approach

**Figure 3:**
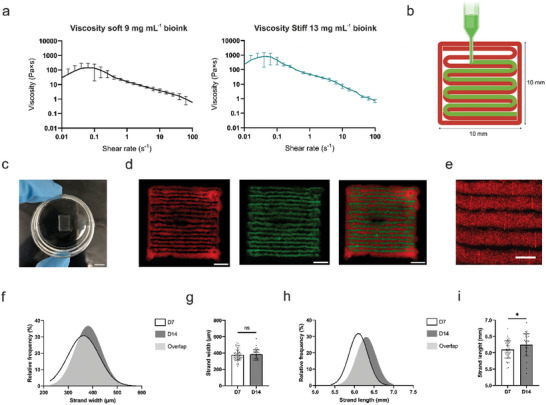
Characterization of the printed structure. a) Viscosity curves of soft and stiff bioinks showing excellent shear‐thinning properties (*n* = 4 for both). b) Schematic illustration of the 3D bioprinting process. Red indicates stiff bioink, and green indicates soft bioink (created with Biorender.com). c) Photograph of the 3D printed structure after printing. d) 5 × magnification representative images of the 3D printed structure. The stiff bioink was supplemented with red fluorescent particles, and the soft bioink was supplemented with green fluorescence particles. Scale bars are 2 mm. e) Representative image of the strand width measurement; red lines indicate the regions of interest (ROIs) used to measure the strand width. Scale bar 500 µm. f,g) Strand width analysis. Frequency distribution and column chart (g: mean ± SD. as whiskers) of the strand width measured on EDIV7 and EDIV14 (*n* = 60). h,i) Strand length analysis. Frequency distribution and column chart (i: mean ± SD. as whiskers) of the strand width measured on EDIV7 and EDIV14. An unpaired *t*‐test was performed to compare the strand width and length between EDIV7 and EDIV14 (g,i). Statistical significance is denoted as ^*^ *p* ≤ 0.05.

The axon guidance structure was prepared via extrusion‐based 3D printing in a multimaterial manner using soft and stiff bioinks introduced in the previous sections. The original composition of the HA‐DA bioink and its printing properties, shape fidelity, and self‐healing properties have been described previously.^[^ ^32^ ^]^ The stiffnesses used in the printing of the axonal guidance structure were chosen based on the optimization of the printing process and following cell growth properties (Table S1, Supporting Information). The viscosity curves revealed differences in viscosity (**Figure** **3**a). The shapes of the curves also indicate excellent shear‐thinning properties, which are crucial factors for the used bioinks in extrusion‐based 3D bioprinting and 3D printing.

Figure 3b shows the 3D printing of the first layer of the axon guidance structure. It contains both stiff and soft bioinks as alternating continuous strands surrounded by contours made with stiff bioink. The structure of six identical layers was successfully 3D printed, in which stiff bioink formed a cell guiding structural support and soft bioink provided tunnels for axonal innervation through the structure (Figures 3b,7). Based on the results from Section 2.1., soft bioink was supplemented with NGF, and increased concentration of laminin.

The initial theoretical dimensions of the axon guidance structure were set to 10 mm × 10 mm × 480 µm after 3D printing (Figure 3c) and 6 mm × 10 mm × 480 µm after the edges were cut. The soft strands filled the space between the stiff strands and the structure maintained its shape excellently after the printing (Figure 3d) and further in cell culture conditions up to EDIV14. To analyze the stability of the axonal guidance structure, the strand width and length of the stiff bioink strands were measured. The frequency distribution histograms of the strand width on EDIV7 and EDIV14 showed good overlapping curves, and the average strand width increased only slightly from 377 ± 66 µm to 385 ± 56 µm within one week (Figure 3g). Strand length was influenced by the preparation of the guidance structure, that is, the successful cutting of the edges with a mold. Nevertheless, the strand length also showed overlapping distribution curves, with the length increasing from 6.10 ± 0.26 mm to 6.25 ± 0.34 mm (Figure 3h). The tunnel length was derived from the strand length, i.e., an average of the strands lining a certain tunnel. A similar increase in tunnel length compared with strand length was observed (Figure 3i).

### 2.4 Axonal Growth toward the Soft Tunnels of the Guidance Structure

**Figure 4:**
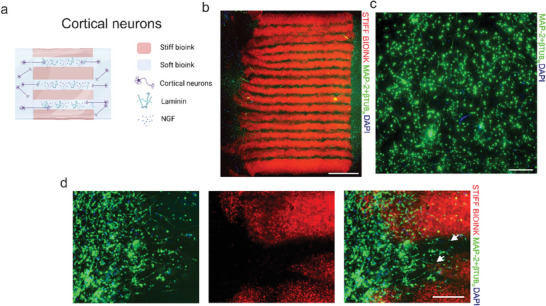
Cortical neurons in the 3D printed structure. a) Schematic top view of the 3D printed guidance structure with the neurons (created with Biorender.com). b) 5 × magnification fluorescence image of the 3D printed guidance structure with cortical neurons. Scale bar 2000 µm. c) 20 × magnification of cortical neurons at the edge of the axon guidance structure. Scale bar 200 µm. d) Close‐up image of the interface of the axon guidance structure and the cell bioink. The arrows indicate ingrown axons. Scale bar 200 µm, 20 × magnification. Neurites are stained with MAP‐2+βtub_III_ (green), nuclei are stained with DAPI (blue), and stiff bioink is shown in red.

In the initial experiments, the ability of HA‐based 3D printed axon guidance structures to allow innervation was evaluated by utilizing cortical neurons as used in bulk bioink experiments. A top‐view illustration of the axon guidance structure and neurons plated in the cell bioink at the edges of the structure is shown in **Figure** **4**a. The structures were assembled successfully (Figure 4b). The mold used to cut the edges caused some variation in the structure, but it did not affect the plating of the cells, as neurons formed a good network at the edges of the structure (Figure 4c). Cortical neurons innervated the structure only to a minor extent although the final structure showed good cell viability and structural stability up to EDIV14(Figure 4d).

**Figure 5:**
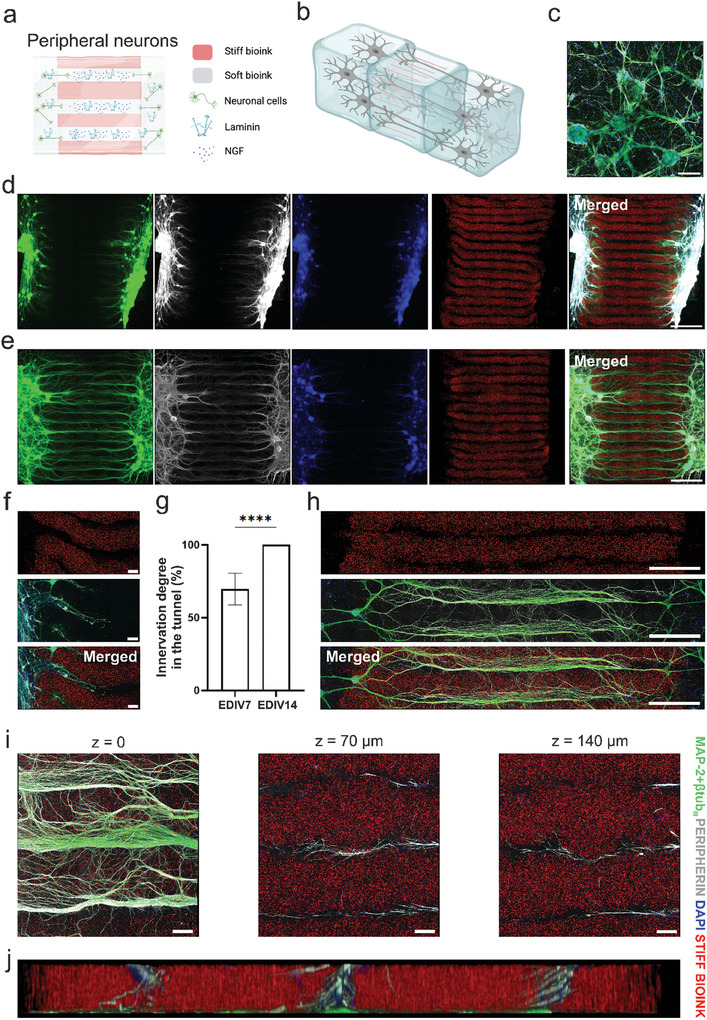
Peripheral neurons innervate the axon guidance structure. a) Schematic 2D top view of the structure and b) 3D illustration of the axon guidance structure with the neurons (created with Biorender.com). c) Neurons formed networks in soft bioink at the edge of the structure (20 × magnification, scale bar 200 µm). d) 5 × magnification fluorescence image of the whole structure on EDIV7 and e) EDIV14. Scale bars 2000 µm f) Close‐up details from EDIV7 and h) EDIV14 (10 × magnifications, scale bars 200 and 1000 µm, respectively). g) Innervation degree of the axons in the soft bioink tunnels on EDIV7 (*n* = 42) and EDIV14 (*n* = 28). Statistical significance was determined with a nonparametric Mann‐Whitney U test, ^****^ *p* < 0.0001. The data are shown as means + SD. i) Close‐up details from different z‐planes of the structure (EDIV14, 20 × magnification, scale bar 200 µm). j) Side view from the z‐stack showing the axon at every level of the structure. Neurites are stained with MAP‐2+βtub_III_ (green) and peripherin (gray) while nuclei are stained with DAPI (blue) and stiff bioink is shown in red.

Next, peripheral neurons were incorporated into the axonal guidance structure as they possess greater innervation capacity.^[^ ^42^, ^43^ ^]^ A 3D illustration (**Figure** **5**a) as well as a 2D top view (Figure 5b) shows the results schematically. The peripheral neurons formed good networks in the 3D environment when plated at the edges of the axon guidance structure in the cell bioink (Figure 5c). At EDIV7, axons innervated inside the guidance structure and precisely followed the pattern formed by stiff bioink strands (Figure 5d). The axons also followed curve‐shaped tunnels and irregular tunnels, confirming the ability of the stiff bioink to guide the direction of the axons (Figure 5f). At EDIV14, all the tunnels that were continuously filled with soft bioink were fully innervated, and axons growing from both ends formed continuous innervation through the structure (Figure 5e). Details from the samples on EDIV14 are presented in Figures 5h,i. The most densely grown axons were observed at the bottom of the structure since it is the only restriction for axons to grow in the z‐direction. Nevertheless, confocal images revealed that a notable number of axons were also present at the higher levels in z‐stack (Figure 5i,j). The degree of innervation was evaluated by calculating the tunnel length and the longest axon growing inside it. This analysis supports the visual observations, as the average degree of innervation in individual completely open tunnels significantly increased from 69.7% ± 10.9% at EDIV7 to 100% at EDIV14.

## 3 Discussion

Here, we report the development of a sophisticated hydrogel‐based structure for directed 3D axonal growth via an extrusion‐based multi‐material 3D bioprinting approach to model innervation in vitro. The selected hydrogel material used was a previously developed HA‐based bioink.^[^ ^32^, ^34^ ^]^ It has an ideal composition for neuronal applications because of its resemblance to the natural ECM composition of brain tissue^[^ ^13^, ^14^ ^]^ as collagen and HA‐based hydrogels mimic the soft and elastic nature of the brain ECM.^[^ ^32^, ^44^, ^45^ ^]^ Although innervation in this study was achieved with human peripheral neurons, human cortical neurons were utilized for optimizing the printing process, as they were successfully used previously in an innervated corneal model.^[^ ^32^ ^]^

### 3.1 NGF and Laminin Increase the Neuronal Network Formation in 3D Cultures

External molecules that resemble native guidance cues in vivo are needed to achieve controlled axonal guidance in vitro. In vivo, collagen, laminin, and different neurotrophic factors are upregulated after injury promoting axonal guidance, cell adhesion, and cell survival.^[^ ^41^ ^]^ Here, NGF and laminin were used in soft bioink to increase the growth of and guide innervating axons in the 3D bioprinted structures, both of which have been shown to promote neurite growth in vitro.^[^ ^41^, ^46^ ^]^ To evaluate the effects of NGF and laminin on network formation in 3D, total network length and network volume were chosen as parameters for quantification via image‐based analysis. Additionally, the connectedness of the 3D neuronal network in different groups supplemented with NGF, laminin, or both were evaluated, as disconnections in the networks can indicate damage.^[^ ^47^ ^]^ Compared with other groups, the NGF+laminin 10 m presented increased total network length and volume. Moreover, higher cell density (10 M) resulted in longer network formation and greater network volume compared to samples with lower cell density (5 m). NGF+laminin also increased the network connectedness, as the mean volume fraction of the largest connected component was >90% at both cell concentrations and reached 98% at higher cell density. The most disconnected network was observed with NGF 10 m. Thus, neither NGF nor laminin alone promoted connectedness as well as their combination. Previously, NGF and laminin have been shown to improve 3D neuronal network formation or neurite extension separately with human cortical neurons and with rat dorsal root ganglion (DRG) neurons,^[^ ^40^, ^41^ ^]^ and laminin with hiPSC‐derived neurospheres.^[^ ^48^ ^]^ However, there seems to be favorable crossplay between NGF and laminin as shown in a study using their gradients in agarose hydrogels.^[^ ^49^ ^]^ Furthermore, the increased secretion of NGF from rat Schwann cells in the presence of laminin in collagen and HA hydrogels has also been reported^[^ ^50^ ^]^ supporting the crossplay of NGF+laminin with human neurons. The supplementation of NGF+laminin with 10 m cells was chosen for future experiments, as the trend in total network length and volume also indicated a positive effect on neuronal network formation.

### 3.2 Bioink Stiffness Affects the Formation of a 3D Neuronal Network

The stiffness of bioinks is widely adjustable by altering the concentration of the crosslinking components. Here, two modifications of HA‐DA bioink^[^ ^32^ ^]^ with different stiffnesses were used, a soft bioink with a concentration of 9 mg mL^−1^ and a stiff bioink with a concentration of 13 mg mL^−1^ crosslinking components. The mechanical properties of the bioinks, including viscoelastic behavior, stability, and shear thinning profile, were characterized via rheological measurements. Both the loss modulus and the storage modulus were greater with the stiffer bioink (12.7 ± 6.9 Pa and 206.2 ± 19.0 Pa, respectively) than with the soft bioink (6.1 ± 1.1 Pa and 71.0 ± 7.3 Pa, respectively), indicating a clear difference in their mechanical strength. Moreover, both bioinks can be considered viscoelastic materials since G′ > G″ at all frequencies. The stiffness of the CNS varies depending on the anatomical location, individual's age, and pathological condition, with a range of shear modulus between 0.1 and 10 kPa.^[^ ^51^ ^]^ As expected, neuronal growth was good in the soft bioink and restricted in the stiff bioink, which was further supported by previous in vitro studies demonstrating the preference for mechanically softer hydrogels for neurons.^[^ ^52^, ^53^ ^]^ Moreover, an increase in neuronal stem cell proliferation and the upregulation of βtub_III_ markers have been shown in soft hydrogels with a modulus of < 200 Pa compared with stiffer hydrogels.^[^ ^54^ ^]^ Currently, implanted TE constructs lack preformed neuronal connections,^[^ ^32^ ^]^ highlighting the need for biomaterials that mimic the mechanical properties of the transplantation site to support neuronal growth both in vitro and further post‐transplantation in vivo.^[^ ^3^ ^]^

### 3.3 Novel 3D Axonal Guidance Structure with Multimaterial Printing

To better mimic complex neuronal tissue in vivo, more sophisticated models with hydrogel‐based TE structures are needed. Here, extrusion‐based multi‐material 3D bioprinting was used with two separate cartridges and printing heads for both bioinks, allowing the precise deposition of biomaterials to the 3D axonal guidance structure. The bioinks were extruded through a 100 µm needle, close to the limit of extrusion‐based bioprinting,^[^ ^55^ ^]^ to achieve the highest possible resolution of the guiding structure. The printed structure was followed for 14 days, and it maintained its shape with distinguishable strands of both bioinks with and without the cells. The stiff bioink maintained its original shape during the culturing period, however, a small variance between the strand lengths was observed most likely due to human error during the removal of the edges of the structure. As the stiff bioink has better shape fidelity properties, it was used to characterize the shape of the structure. The follow‐up time of 3D bioprinted in vitro models for neuronal guidance varies, commonly being up to 14 days.^[^ ^56^, ^57^, ^58^, ^59^ ^]^ However, systems created with similar extrusion bioprinting technology, as high resolution and cell‐friendly chemically crosslinkable bioink without any additional crosslinking methods, e.g. UV‐light could not be found in the literature.

As neurons favor mechanically soft hydrogels,^[^ ^52^, ^53^ ^]^ soft bioink was used to create guiding tunnels for axons within the structure. The interface between the soft and stiff bioink strands offers biophysical guidance that was further supported by incorporating NGF and laminin into the soft bioink passages as biochemical cues. Although extrusion‐based bioprinting has been used for creating neuronal 3D bioprinted models,^[^ ^32^, ^57^, ^60^ ^]^ to the best of our knowledge, 3D multimaterial structures similar to those presented here have not been created to guide axons in vitro. Moreover, the current 3D bioprinting technology lacks efficiency in enabling the orientation of mature human neurons crucial for ensuring physiologically more accurate in vitro tissue models^[^ ^61^ ^]^ for which we provide a novel, successful solution.

### 3.4 Successful 3D Axonal Guidance

Human cortical neurons were used to establish this model, since the cytocompatibility of the original bioink with these cells has been shown with successful innervation of the cortical neurons into 3D bioprinted cornea stromal structures.^[^ ^32^ ^]^ Here, the same HA‐DA bioink with slight improvements was used. The cortical neurons were added to the edges of the printed structure, where the cells remained viable for 14 days. However, the axons did not grow into the structure despite the formation of a neuronal network at the edges of the structure. Owing to the preparation process of the complete cell, including the model, an interface may form between the axon guidance structure and the cell bioinks. According to previous results,^[^ ^34^ ^]^ however, it is expected that the addition of NGF and laminin would have enhanced the ingrowth of the axons into the soft bioink tunnels. Compared with previous cornea models,^[^ ^32^, ^40^ ^]^ the lack of other cell types in the structure may have lessened the innervation potential of the cortical axons. When a similar setup with hPSC‐derived peripheral neurons was used, successful innervation through the soft bioink into the structure was clear already on EDIV7. On EDIV14, all the soft bioink passages in the printed structure were fully innervated by the peripheral axons. Peripheral neurons have a natural ability to grow longer and thicker axons and innervate tissues,^[^ ^3^ ^]^ whereas cortical neurons tend to form network‐like structures.^[^ ^62^ ^]^ Enhancing neuronal network formation and guiding axonal growth are separate phenomena, and different neuronal subtypes respond differently to different chemoattractants.^[^ ^15^ ^]^ Thus, when innervation and axonal guidance are considered, the selection of an appropriate neuronal type is crucial to ensure the physiological relevance of the model.

Different 2D platforms for studying and modeling innervation in vitro exist.^[^ ^63^, ^64^, ^65^ ^]^ However, the 3D environment provides cells with a physiologically more relevant microenvironment, allowing spatial cell elongation, cell‐to‐cell, and cell‐to‐ECM interactions. Our model successfully connects two separate neuronal populations in 3D, without the need for compartmentalized, artificial structures such as microfluidic chips.^[^ ^66^ ^]^ The printed structure offered control over the orientation in the x‐y direction but not in the z‐direction. Thus, axons grew in an arbitrary manner in the x direction. The neuronal soma aggregates dispersed throughout the height of the cell ink next to the guidance structure, but most dense axonal growth was detected close to the bottom of the dish, i.e., at the plastic‐bioink interface. In the future, additional restrictions of stiff bioink layers could be tested to increase the even distribution of axons in the z‐direction.

Our developed 3D axon guidance model offers many possibilities for neuronal TE with directed axonal organization. It has great potential for studying axon growth and elongation, the formation of axon tracts and nerves in different locations of the nervous system, and the impact of different biochemical molecules on axonal growth. Additionally, modeling more complex phenomena, such as axonal damage or injury and innervation of target tissue in both the CNS and PNS, is possible. Compared with nontransparent hydrogels^[^ ^57^ ^]^ or hard melt‐processed polymers,^[^ ^55^ ^]^ the structure consists of only transparent hydrogel‐based bioinks, enabling easy visualization of neurite growth during culture. Easy access to the hydrogel structure also enables the induction of axonal injury with different physical, chemical, and mechanical factors. The model is not restricted only to these certain neurons or cell types in general since it can be easily modified. The manufacturing process is not harsh to the cells because of chemical crosslinking without toxic crosslinking agents or photocurable bioink.^[^ ^32^ ^]^ Soft bioink in the guidance structure can be supplemented with a variety of bioactive molecules compatible with the cell type used. Thus, this model has great potential as a stand‐alone platform for axonal studies or as an added part for more complex organ/body‐on‐chip platforms.

## 4 Conclusion

Here, we developed a novel and robust 3D axonal guidance structure with multimaterial 3D bioprinting and hPSC‐derived neurons. Our approach utilizes two acellular HA‐based bioinks with different stiffnesses bioprinted as alternating, parallel‐aligned filaments. The soft passages, which incorporated the attractant cues NGF+laminin, facilitated directed axonal growth. The structures maintained their shape throughout the experiments with or without cells for up to 14 days. The soft and stiff bioinks presented different loss and storage modulus as well as shear thinning properties. Moreover, the soft bioink supplemented with NGF+laminin supported 3D neuronal network formation better than the stiff bioink supporting the hypothesis that axons preferably grow through the soft bioink. Both cortical and peripheral neurons formed viable 3D networks at the edges of the axonal guidance structure. On day 7, peripheral axons from both sides of the structure had clearly elongated into the soft passages and on day 14 all the soft passages were fully innervated forming bidirectional connections.

Innervation is often overlooked in TE applications despite its pivotal role in the development, maturation, and function of tissues both in health and disease, highlighting the need for novel 3D in vitro innervation models. The present work provides a robust 3D axonal guidance model that can be used to model 3D innervation in vitro. The model has the potential for expanded usage in drug screening and disease modeling as well as more complex TE models with additional cell and tissue types.

## 5 Experimental Section

### Bioink—*Preparation of the Bioinks*

Conventional hydrazone crosslinking chemistry was used with the HA‐dopamine (DA) bioink. The crosslinking components of HA‐DA bioink were HA‐DA‐carbodihydrazide (CDH) and HA‐aldehyde (HA‐ALD). The synthesis of the crosslinking components was performed according to previously published protocols.^[^ ^67^, ^68^ ^]^ The materials were lyophilized after synthesis and stored at −20 °C until further use.

**Figure 6:**
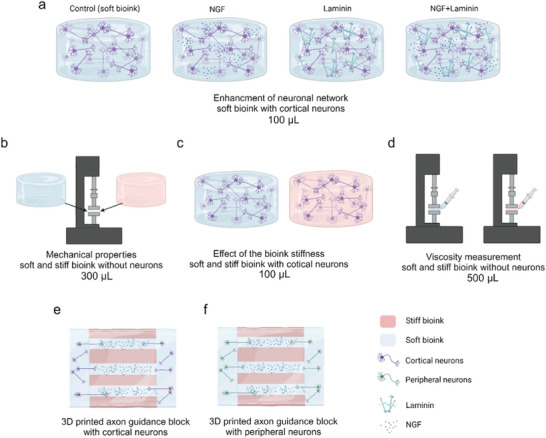
Different experimental settings where bioinks were used. a) Cortical neurons (5 and 10 m) were cultured in 4 different soft bioink conditions: control and cultures supplemented with NGF, laminin, or NGF+laminin. b) The mechanical properties, i.e., loss and storage modulus of soft and stiff bioinks were studied. c) The effect of the stiffness of the bioink on cortical neuronal network formation was evaluated and d) the viscosity of the soft and stiff bioinks was measured. e) Cortical neurons and f) peripheral neurons were used in the 3D axonal guidance model with multimaterial 3D printed axon guidance structure. Created with Biorender.com.

The HA‐DA bioink used in this study was prepared as previously published^[^ ^32^ ^]^ with slight modifications. Here, a multimaterial printing approach was used with three different modifications of the bioink, referred to as stiff bioink, soft bioink, and cell bioink, to introduce axonal guidance in 3D printed structures. For the stiff bioink, the crosslinking components were diluted in 1× PBS (Dulbecco's Phosphate Buffered Saline, DPBS, Carl Roth, Germany) into 13 mg mL^−1^ concentration. For visualization of the stiff bioink strands, FluoSphere fluorescent nanoparticles (ø = 0.5 µm, excitation/emission wavelength red 580/605, Invitrogen, USA) were added to the bioink with 0.1 V% of the whole bioink. In soft bioink, crosslinking components were diluted to 9 mg mL^−1^ concentration. OptiCol Human Collagen Type I (Cell Guidance Systems, UK) was added to the bioink for improved cytocompatibility and as a rheological modifier. It was neutralized to pH 7.4 using 1 m NaOH and 10× DPBS (Carl Roth, Germany). In addition to the original composition of the bioink, the soft bioink was supplemented with 3.8 µg mL^−1^ LN521 (BioLamina, Sweden), 400 ng mL^−1^ NGF (R&D Systems, USA) or 1 mg mL^−1^ mouse laminin (L2020, Sigma‐Aldrich, USA). Moreover, for visualization of the soft bioink strands, FluoSphere fluorescent nanoparticles (ø = 0.5 µm, excitation/emission wavelength yellow/green 505/515, Invitrogen, USA) were added with a 0.1 V‐% of the whole bioink. Finally, for the cell‐containing bioink, the crosslinking components were diluted into the 9 mg mL^−1^ concentration and supplemented with 3,8 µg mL^−1^ LN521. The cell culture medium used to prepare the bioinks for the experiments including cortical neurons was reduced neural nedium (RPM), as described in the Experimental Section, and for peripheral neurons sympathoadrenal maturation medium, as described in the Experimental Section, was used. The different experimental setups using soft and/or stiff bioink included: studying the effects of NGF and laminin on cortical neuron network formation in 3D soft bioink cultures (**Figure** **6**a), the mechanical properties of the bioinks through loss and storage modulus (Figure 6b), the effect of the stiffness of the bioink on 3D cortical neuron network formation (Figure 6c), the viscosity of the bioinks (Figure 6d) and the guided 3D axonal innervation of cortical neurons (Figure 6e) and peripheral neurons (Figure 6f) in the 3D printed axon guidance structure.

### Bioink—*Mechanical Properties – Loss and Storage Modulus*

The stiffness of the bioinks was analyzed by measuring their mechanical properties separately from gel disks (volume 300 µL). After preparation, the bioinks were distributed into cut syringes (Ø 12 mm) and crosslinked for 3 h. Then, the gel discs were transferred to 24‐well plates, covered with 1× PBS, and incubated at 37 °C. Amplitude and frequency sweeps were measured with an HR‐II Discovery hybrid rheometer (TA Instruments, USA) after 72 h using a 12 mm parallel plate geometry. The gap between plates was manually set to 1.3 mm. Amplitude sweeps were carried out with a constant frequency of 1 Hz and oscillation strains ranging from 0.01% to 100%. Three replicates were measured during the amplitude sweep. Frequency sweeps were carried out with frequencies ranging from 0.1 to 10 Hz and with a constant strain of 10% based on the amplitude sweeps.

### Bioink—*Shear Thinning – Viscosity*

The shear‐thinning properties of the bioinks were determined by measuring the viscosity with an HR‐II Discovery hybrid rheometer. The prepared bioinks were allowed to pre‐crosslink immediately and measured with a 20 mm parallel plate geometry at 20 °C under a continuous flow rate with a shear rate ranging from 0.01 to 100 s^−1^. The samples were produced in a 500 µL volume with a gap manually set to 1 mm.

### Neuronal Cells (Origin and Differentiation)—*Cortical Neurons*

Cortical neurons were differentiated from the human embryonic stem cell (hESC) line Regea 08/023^[^ ^69^ ^]^ using a previously published protocol.^[^ ^70^ ^]^ Briefly, the neural maintenance medium consisted of 1:1 DMEM/F12 with GlutaMAX and Neurobasal, 0.5% N2, 1% B27 with retinoic acid, 0.5 mm GlutaMAX, 0.5% NEEA, 50 µm 2‐mercaptoethanol (all from Thermo Fisher Scientific, USA), 2.5 µg mL^−1^ insulin (Sigma‐Aldrich) and 0.1% penicillin/streptomycin (Thermo Fisher Scientific). In the induction stage of differentiation, at 1–12 days in vitro (DIV), the medium was supplemented with 100 nm LDN193189 (Sigma‐Aldrich) and 10 µm SB431542 (Sigma‐Aldrich), at the neural proliferation stage (DIV13–25), the medium was supplemented with 20 ng mL^−1^ fibroblast growth factor‐2 (FGF2, Thermo Fisher Scientific) and at the final maturation stage (DIV26–32), the medium was supplemented with 20 ng mL^−1^ brain‐derived growth factor (BDNF, R&D Systems), 10 ng mL^−1^ glial‐derived neurotrophic factor (GDNF, R&D Systems), 500 mm dibutyryl cyclic adenosine monophosphate (db‐cAMP, Sigma‐Aldrich) and 200 mM L‐ascorbic acid (AA, Sigma‐Aldrich) referred to as neural maturation medium (NMM) hereafter. On DIV32, the neurons were detached with StemPro Accutase (Thermo Fisher Scientific) and seeded in 6‐well plates for further maturation, and on DIV39 cells were plated for the final experiments. To differentiate between the DIVs referring during neuronal differentiation and during the experimental phase, the term experimental days in vitro (EDIV) is used to refer to DIVs after the differentiation.

For the final experiments, the neurons were detached with StemPro Accutase, centrifuged, resuspended in culture medium, and mixed in HA‐DA bioink at a density of 5 × 10^6^ cells mL^−1^ (5 m) or 10 × 10^6^ cells mL^−1^ (10 m). After the cells were moved into the 3D environment, the RPM containing neurobasal medium supplemented with 0.5% N2 supplement, 0.5 mm GlutaMAX, 0.1% penicillin/streptomycin, 20 ng mL^−1^ BDNF, 10 ng mL^−1^ GDNF and 500 µm db‐cAMP was used as the culture medium as published previously.^[^ ^32^ ^]^ All the cultures were maintained at 37 °C in a 5% CO_2_ and 95% humidity.

### Neuronal Cells (Origin and Differentiation)—*Peripheral Neurons*

The differentiation of sympathetic neurons (SNs) from hPSCs was adapted from a published protocol.^[^ ^71^ ^]^ The hPSC line used for the differentiation of SNs was the in‐house‐derived and characterized hiPSC line 0 4511.WTs.^[^ ^72^ ^]^ Briefly, neuromesodermal progenitor (NMP) inducing medium consisted of DMEM/F‐12 without glutamine and neurobasal medium supplemented with 1× B‐27, 1× N2, 1× GlutaMAX, 1× NEAA, 100 µm 2‐mercaptoethanol, 1× penicillin/streptomyosin, 4 µm CHIR99021 (Axon MedChem) and 20 ng mL^−1^ bFGF (R&D Systems) until DIV3. On DIV3, NMPs were switched to neural crest (NC) medium containing DMEM/F‐12 without glutamine supplemented with 1× N2, 1× GlutaMAX, 1× NEAA, 0.1× penicillin‐streptomycin, 2 µm SB431542 (Sigma‐Aldrich, USA), 1 µm CHIR99021, 1 µm DMH1 (Tocris, UK) and 15 ng mL^−1^ recombinant human BMP4 (Peprotech, UK) for trunk NCs differentiation phase until DIV8. On DIV8, the media was changed into the sympathoadrenal medium consisting of BrainPhys (StemCell Technologies) supplemented with 1× B27, 1× N2, 1× GlutaMAX, 1× NEEA, 0.1× penicillin‐streptomycin, 50 ng mL^−1^ Sonic Hedgehog C24II (R&D Systems), 1.5 µm Purmophamine (Sigma Aldrich) and 50 ng mL^−1^ Recombinant Human BMP4. On DIV12, the media was changed into sympathoadrenal maturation medium consisting of BrainPhys with 1× B27 1× N2, 1× GlutaMAX, 1× NEEA, and 0.1× penicillin‐streptomycin, supplemented with 10 ng mL^−1^ BDNF, 10 ng mL^−1^ GDNF and 10 ng mL^−1^ NGF (R&D Systems). Medium changes were performed daily for 4 days, and on DIV23 SNs were detached into the 3D printed cultures as cortical neurons at a density of 10 m mL^−1^ in sympathoadrenal maturation medium. SNs expressed adequate morphology and typical SN‐markers on DIV23, indicating successful differentiation and maturation of the cultures (Figure S1, Supporting Information).

### Ethical Statement

The hPSCs used in this study were acquired from voluntary subjects who provided written and informed consent. The project has a supportive statement from the Ethics Committee of the Expert Responsibility Area of Tampere University Hospital to use the named hPSC lines in neuronal research (R20159).

### 3D Bulk Bioink Cultures

The possibility of enhancing 3D neuronal network formation in 3D bulk bioink cultures was studied using 5 and 10 m cortical neurons. The control group did not contain any of the supplements, and a total of 4 groups/cell concentrations were generated (Figure 6a). The cortical neurons were mixed with bioink, and after 15 min of pre‐crosslinking at RT, the bioink‐cell samples were plated on a glass Mattek 24‐well plate (Mattek) in a volume of 100 µL followed by 30 min of crosslinking at 37 °C before the medium. The samples were cultured for 14 days in RPM.

### 3D Bioprinting of the Axonal Guidance Structure

An extrusion‐based 3D‐Bioplotter Manufacturer Series by EnvisionTEC (Gladbeck, Germany) 3D bioprinter with a low‐temperature printing head suitable for the 3D bioprinting of hydrogel‐based materials was used with the temperature adjusted to 20 °C. The bioink was transferred to a 30‐cc barrel (Nordson EFD, USA) after mixing and allowed to precrosslink at RT (45 min for soft bioink and 15 min for stiff bioink). 32 G (inner Ø 100 µm) blunt needles 0.50 inches in length (CellInk, USA) were used for printing.

The preparation of the axon guidance structure is illustrated in Figure 1. Each layer had a contour using the stiff bioink with dimensions of 10 mm × 10 mm, followed by the printing of inner alternating stiff and soft bioink filaments as continuous strands. The stiff bioink was printed first, and then, the soft bioink was further printed to fill the gaps between the stiff filaments. The distance between the strands was 0.6 mm for both bioinks. The structure consisted of six similar layers. Printing was performed at RT according to the 3D model in STL format created with Perfactory RP software (EnvisionTEC, USA) using an 80 µm slicing interval. The inner structures of both bioinks for each layer in the printed structure were determined with Visual Machines software, with which a 3D bioprinter was also used. The printing window for both bioinks was ≈1 h, and the parameters for soft and stiff bioinks during this time window were as follows: pressures of 0.7 bar and 2.3 bar, speeds of 15.1 mm s^−1^ and 19.0 mm s^−1^, preflows of 0 s and 0.4 s, respectively. Plastic Petri dishes (35 mm) were used as printing substrates (plasma treated, Corning, USA).

**Figure 7:**
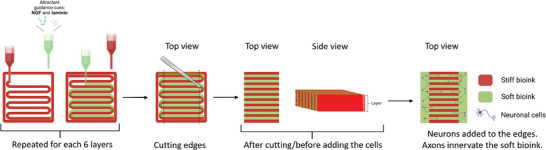
Schematic illustration of the preparation of the axon guidance structure. Created with Biorender.com.

Printing was followed by a 30 min stabilization period at 37 °C, after which the opposing edges of the axon guidance structure, perpendicular to the guiding tunnels, were manually removed using a 6 mm wide rectangular metal mold and spatula. The cells, either cortical neurons or peripheral neurons, were mixed with the additionally prepared soft bioink (cell bioink, see Section 2.1.2) and pipetted to the opposing sides of the structures (50 µL), which was followed by another 30 min incubation at 37 °C before the addition of the cell culture media. The printing workflow is shown in **Figure** **7**.

### Immunocytochemical Staining

Immunocytochemical (ICC) staining was performed via previously published protocols for 3D samples.^[^ ^24^, ^41^ ^]^ In brief, 3D bulk bioink samples were fixed on EDIV14, and 3D printed samples on EDIV7 and EDIV14 with 4% PFA in PBS for 1 h at RT. 10% normal donkey serum (NDS, Millipore, Germany), 0.1% TritonX‐100, and 1% bovine serum albumin (BSA, Sigma‐Aldrich) in PBS were used to block the nonspecific antigen binding sites for 60 min at RT. The samples were incubated with primary antibody solution for three days at 4 °C. The following primary antibodies were used: β‐tubulin III (βtubIII, rabbit, 1:250, A01627, GenScript, USA), βtubIII (rabbit, 1:100, ab52623, Abcam, UK), microtubule‐associated protein 2 (MAP‐2, rabbit, 1:200, AB5622, Millipore), MAP‐2 (chicken, 1:200, NB300‐213, Novus, USA), neurofilament‐H (NF‐H, mouse, 1:500, N5389, Sigma‐Aldrich), peripherin (PRPH, rabbit, 1:400, AB1530, Millipore). Then, secondary antibodies were incubated for 24 h at 4 °C. Following Alexa‐labeled secondary antibodies were used: goat anti‐chicken 488 (A11039, 1:200), donkey anti‐rabbit 488 (A21206, 1:200), donkey anti‐mouse 568 (A10037, 1:200), goat anti‐chicken 647 (A21449, 1:200), donkey anti‐mouse 647 (A31571, 1:125), donkey anti‐rabbit 647 (A31573, 1:200) (all from Thermo Fisher Scientific). A 1:4000 dilution of DAPI (Sigma‐Aldrich) was used with secondary antibodies. Finally, the samples were mounted with Vectashield Antifade Medium (Vector Laboratories, USA) and stored light‐protected at 4 °C. Images were processed with ImageJ Fiji software (U.S. National Institutes of Health, version 1.54f). And LasX software (Leica, Germany).

### Imaging

An LSM780 Laser Scanning Confocal Microscope (Carl Zeiss, Germany) with a 25× glycerin immersion objective (numerical aperture, NA 0.8) was used for confocal imaging of 3D bulk bioink samples, and an LSM800 Laser Scanning Confocal Microscope (Carl Zeiss) with a 10× air immersion objective (NA 0.3) was used for imaging of 3D printed axon guidance structures. For fluorescence imaging of 3D printed structures, a DMi8 inverted microscope (Leica) was used.

### Analysis—*Bulk Bioink, Enhancing the Formation of the Neuronal Network*

To study the properties of the neuronal network in 3D bulk bioink samples, 128 images from 2 different experiments were analyzed (*n* = 16 per group). All the images had a resolution of 0.33 × 0.33 × 0.5 µm, and image stacks were 325 × 325 × 100 µm in size. 3D image stacks were deconvoluted with Huygens Essentials software (Scientific Volume Imaging). Neurite tracing and analyses were conducted with Imaris software (Oxford Instruments, UK) according to previously published methods.^[^ ^45^ ^]^ The neurite channel was first merged with the DAPI channel via the MATLAB (version 2022b) “channel arithmetic” function and smoothed with a Gaussian filter (0.332). For the filament tracing analyses, 1 or 2 µm seed‐point diameter, i.e., the neurite diameter, 12 µm starting‐point diameter, and 4 µm maximum gap width was used. To calculate the neurite volume, a surface tool was used by adjusting the surface detail to 0.6. The automatic mode of the Autopatch algorithm was subsequently used for neuronal tracing.

### Analysis—*Characterization of the Printed Structure*

The 3D printed material was characterized at EDIV7 and EDIV14 to determine the reproducibility of the 3D printing technique and its structural stability during the cell culture period. For that purpose, the strand width and strand length were measured. The height of the 3D‐printed axon guidance samples was approximately 300 µm (z‐stack).

For the strand width analysis, one focus plane between the bottom and maximum height of the sample was chosen for all analyzed structures separately, and the strand widths were measured from that certain layer. In this way, the whole z‐stack is represented in the analysis. For one strand value, 3 different points were measured within a strand and averaged. In total, 10 strands per sample were analyzed, and 6 samples from three separate experiments at time point EDIV7 and 4 samples from two experiments at time point EDIV14 were used for calculating the strand width. In total 60 or 40 analyzed strands were obtained. Strand width analysis was performed using ImageJ Fiji software.

The innervation degree was analyzed from structures with peripheral neurons. The degree was calculated from individual soft tunnels. First, the qualified tunnels from each sample were chosen, which was determined as one region of interest (ROI). Each tunnel was lined with two stiff bioink strands. The lengths of the strands were measured, and the tunnel length was determined to be the average of the strand lengths. This was followed by measuring the longest axon inside this tunnel starting from both sides of the tunnel and summing (Figure S2, Supporting Information). Because stiff bioink strands were above each other in the guidance structure, the growth of axons was not restricted in the z‐direction except at the bottom of the dish. Hence, for innervation degree analysis, one focus plane could not be chosen. However, in most cases, the longest axon in the tunnel was situated near the bottom of the sample. The innervation degree was calculated as the proportion of the tunnel length filled with axons (Equation 1).

(1) $$\begin{array}{ccc} Innervation\;degree & = & \frac{Sum\;of\;the\;longest\;axons\;in\;the\;tunnel\;from\;each\;side}{Total\;length\;of\;the\;tunnel}\\ & & \times \;100\% \end{array}$$

In total, 14 tunnels per sample were analyzed and 3 samples on EDIV7 and 2 samples on EDIV14 were used for calculating the innervation degree. In total,42 and 24 analyzed strands, respectively, were identified. Innervation degree and strand length analyses were performed using Leica Application Suite X software (Leica Microsystems GmbH, Bethesda, version 3.7.6.25997).

### Statistical Analysis

The results are reported as the mean ± standard deviation (SD) and as Tukey box plots for 3D bulk bioink data. Statistical analyses were conducted with IBM SPSS Statistics software (version 29.0, IBM, USA) and GraphPad Prism 9 (version 9.3.1., GraphPad Software, USA). The normality of the data was tested with Shapiro‐Wilk's normality test when *n* < 50 and with Kolmogorov‐Smirnov's test when n > 50. The F‐test was used to compare the variances in normally distributed data. The nonparametric Mann‐Whitney U test was used to compare the total neurite length, average neurite volume, and average volume fraction of the largest connected component (%) between the different cell concentrations whereas the Kruskal‐Wallis test with Dunn's multiple comparisons was used to compare the parameters between different groups in 3D bulk bioink samples (*n* = 16 for each group). The nonparametric Mann‐Whitney U test was used to compare the innervation degree (%) in the soft bioink tunnels between EDIV7 (*n* = 42) and EDIV14 (*n* = 28) in 3D printed samples. The unpaired *t*‐test was performed to compare the strand width and length between EDIV7 and EDIV14 (*n* = 60 for both) in 3D‐printed samples. Statistical significance is denoted as ^*^ *p* ≤ 0.05, ^**^ *p* < 0.01, ^***^ *p* < 0.001, ^****^ *p* < 0.0001.

## Conflict of Interest

Anni Mörö reports a relationship with StemSight Oy that includes equity/stocks. Mörö has patent #PCT/FI2022/050,403 pending to Assignee. Based on the Act on the Right in Inventions in Finland, all authors employed by Tampere University have given all rights to the University and have declared no competing interests. Mörö is a co‐founder/shareholder in StemSight Ltd without any connection to the technology and results reported in this manuscript. The other authors declare no conflict of interest.

## Supporting information



Supporting Information

## Data Availability

The data that support the findings of this study are available from the corresponding author upon reasonable request.
