# Supplementary material for: Hyaluronic Acid‐Based 3D Bioprinted Hydrogel Structure for Directed Axonal Guidance and Modeling Innervation In Vitro
Source: Adv Healthc Mater. 2024 Nov 6;14(1):2402504. doi: 10.1002/adhm.202402504 (PMC11694092; doi:10.1002/adhm.202402504)
Supplement: Supplementary file 1 — Supporting Information [file ADHM-14-0-s001.docx]

Supporting Information

Hyaluronic acid-based 3D printed hydrogel structure for directed axonal guidance and modelling innervation in vitro

Laura Honkamäki, Oskari Kulta, Paula Puistola, Karoliina Hopia, Promise Emeh, Lotta Isosaari, Anni Mörö*, Susanna Narkilahti*

For staining of the 2D sympathetic neuronal cultures (Figure S1), a previously published protocol was used ^[74]^. The cells were first fixed with 4 % PFA in PBS for 15 min followed by 2× 5 min washes with PBS and stored at 4 °C. 10 % NDS, 0.1 % TritonX-100 and 1 % BSA in PBS in PBS was used for 45 min in RT to block the nonspecific antigen binding sites. Similar washing solution as with 3D samples was used for diluting the following primary antibodies: anti-PRPH (rabbit, 1:800), anti-βtubIII (chicken, 1:100, ab41489, Abcam), anti-MAP-2 (chicken, 1:4000, NB300-213, Novus), Homeobox C9 (HOXC9, mouse, 1:50, ab50839, Abcam), Paired Like Homeobox 2B (PHOX2B, mouse, 1:500, Sc-376997, Santa Cruz Biotechnology, USA) and Tyrosine hydroxylase (TH, mouse, 1:800, T1299, Sigma-Aldrich) ON at 4 °C. The next day, samples were washed 2× 5 min with 1 % BSA in PBS followed by an incubation light-protected for 1 hour at 4 °C in 1 % BSA in PBS with following Alexa-labelled secondary antibodies: donkey anti-rabbit 488 (A21206, 1:400), donkey anti-mouse 568 (A10037, 1:400), goat anti-chicken 647 (A21449, 1:200). After incubation, samples were 2× with PBS for 5 min and once with phosphate buffer for 5 min at RT. Nuclei were stained with DAPI.


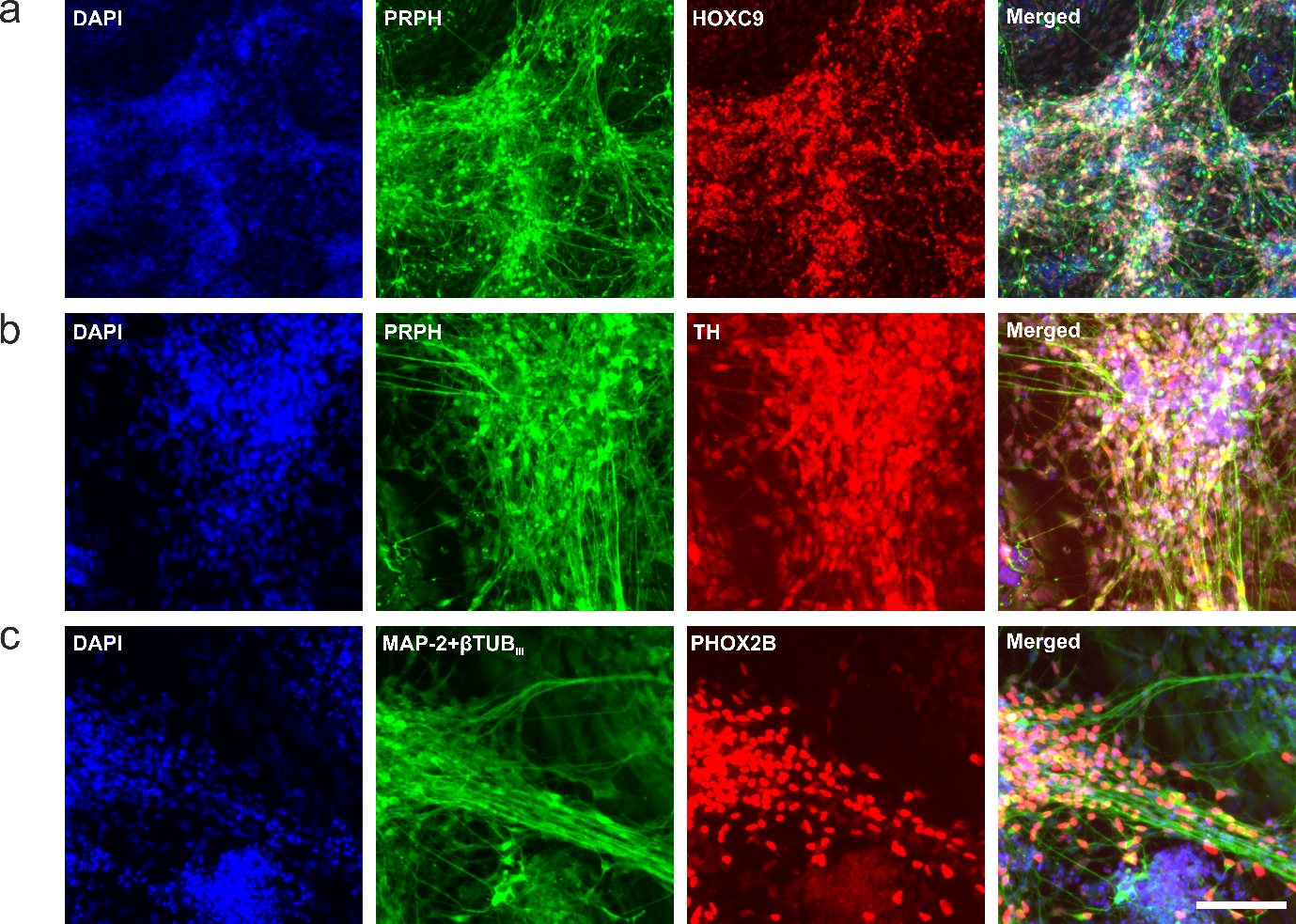


**Figure S1.** Verification of a successful sympathetic neuron differentiation with ICC on DIV23, i.e., the final day of the differentiation and the plating day for experiments. Cultures expressed typical sympathetic neuronal markers, such as a) PRPH and Homeobox protein Hox-C9 (HOXC9) b) PRPH and Tyroxine hydroxylace (TH) and c) MAP-2+ βtubIII and Paired-like homeobox2b (PHOX2B). Nuclei are stained by DAPI. Scale bar is 100 µm and is applicable to all images.


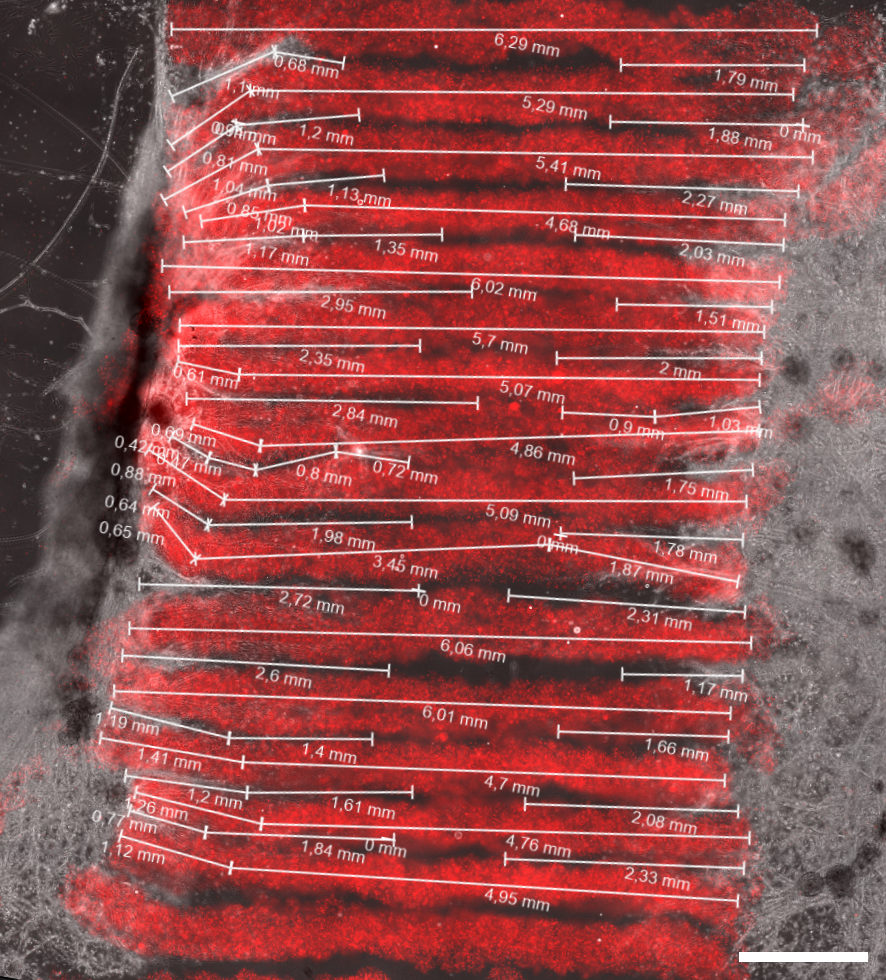


**Figure S2.** Innervation degree analysis. The qualified tunnels from each sample were chosen and this was determined as one ROI. Length of the strands were measured, from which the tunnel length was averaged. The longest axon was measured starting from both ends of the tunnel and summed up. Scale bar in 1000 µm.

**Table S1.** Conducted optimizations in the development phase of the axonal guiding structure.

| **Parameter**  **Bioink** | **Concentration of HA-DA and HA-ALD**  **[mg/mL]** | **Distance between strands**  **[mm]** | **Printing** | | |
| --- | --- | --- | --- | --- | --- |
|  |  |  | **Pressure**  **[bar]** | **Speed**  **[mm s^-1^]** | **Pattern** |
| **Soft** | 9 | 0.5  0.6  0.7  0.8 | 0.4 – 1.0 | 15 - 22 | Continuous strand, separate strands,  with contour,  without contour |
| **Stiff** | 12  13  14 | 0.5,  0.6  0.7  0.8 | 1.0 – 2.5 | 10 - 18 | Continuous strand, separate strands,  with contour,  without contour |
